# Diet-Induced Physiological Responses in the Liver of Atlantic Salmon (*Salmo salar*) Inferred Using Multiplex PCR Platforms

**DOI:** 10.1007/s10126-020-09972-5

**Published:** 2020-06-04

**Authors:** Albert Caballero-Solares, Xi Xue, Beth M. Cleveland, Maryam Beheshti Foroutani, Christopher C. Parrish, Richard G. Taylor, Matthew L. Rise

**Affiliations:** 1 Department of Ocean Sciences, Memorial University of Newfoundland, 0 Marine Lab Road, St. John’s, NL A1C 5S7 Canada; 2 National Center for Cool and Cold Water Aquaculture, ARS/USDA, 11861 Leetown Rd, Kearneysville, WV 25430 USA; 3 Cargill Animal Nutrition, Elk River, MN 55330 USA

**Keywords:** Multiplex PCR, Biomarker genes, Liver, Metabolism, Stress, Inflammation

## Abstract

The simultaneous quantification of several transcripts via multiplex PCR can accelerate research in fish physiological responses to diet and enable the development of superior aquafeeds for farmed fish. We designed two multiplex PCR panels that included assays for 40 biomarker genes representing key aspects of fish physiology (growth, metabolism, oxidative stress, and inflammation) and 3 normalizer genes. We used both panels to assess the physiological effects of replacing fish meal and fish oil by terrestrial alternatives on Atlantic salmon smolts. In a 14-week trial, we tested three diets based on marine ingredients (MAR), animal by-products and vegetable oil (ABP), and plant protein and vegetable oil (VEG). Dietary treatments affected the expression of genes involved in hepatic glucose and lipid metabolism (e.g., *srebp1, elovl2*), cell redox status (e.g., *txna*, *prdx1b*), and inflammation (e.g., *pgds*, *5loxa*). At the multivariate level, gene expression profiles were more divergent between fish fed the marine and terrestrial diets (MAR vs. ABP/VEG) than between the two terrestrial diets (ABP vs. VEG). Liver ARA was inversely related to glucose metabolism (*gck*)- and growth (*igfbp-5b1*, *htra1b*)-related biomarkers and hepatosomatic index. Liver DHA and EPA levels correlated negatively with *elovl2*, whereas ARA levels correlated positively with *fadsd5*. Lower hepatic EPA/ARA in ABP-fed fish correlated with the increased expression of biomarkers related to mitochondrial function (*fabp3a*), oxidative stress (*txna*, *prdx1b*), and inflammation (*pgds*, *5loxa*). The analysis of hepatic biomarker gene expression via multiplex PCR revealed potential physiological impacts and nutrient-gene interactions in Atlantic salmon fed lower levels of marine-sourced nutrients.

**Electronic supplementary material:**

The online version of this article (10.1007/s10126-020-09972-5) contains supplementary material, which is available to authorized users.

## Introduction

The future of farming of Atlantic salmon (*Salmo salar*) and other carnivorous fish lies in the utilization of aquafeeds with a minimum contribution from ingredients sourced in wild fish stocks. Replacement of these marine products—namely, fish meal (FM) and fish oil (FO)—by different terrestrial alternatives has been investigated, with a particular focus on their impact on fish performance and chemical composition (Oliva-Teles et al. 2015; Roques et al. 2018). Growth on experimental feeds with low marine product inclusion has been similar to that with FM/FO-based aquafeeds (Lu et al. 2015; Torrecillas et al. 2017; Beheshti Foroutani et al. 2018). However, the same studies also reported significant changes in the chemical composition of the fish, which could affect the quality of the fish as a commodity. Indeed, omics in fish nutrition research has revealed profound metabolic and physiological adjustments occurring in fish fed diets based on terrestrial products (Sitjà-Bobadilla et al. 2005; Tacchi et al. 2012; Caballero-Solares et al. 2018). Some of the affected physiological functions were related to the welfare and immune status of the fish. For example, the inclusion of plant proteins in the diet increased blood and liver redox status in gilthead sea bream (*Sparus aurata*; Sitjà-Bobadilla et al. 2005) and induced changes in the transcription of immune biomarker genes in the liver of Atlantic salmon (Tacchi et al. 2012; Caballero-Solares et al. 2018). Therefore, as new low-marine aquafeeds are formulated, more investigation will be required to ensure that they promote not only fish growth but also an appropriate health status.

Aquafeed producers test new formulations routinely and need cost-effective methods to evaluate their performance and the physiological effects on the fish, in addition to the measuring of phenotypic features (e.g., weight, length, hepatosomatic index, proximate body composition). High-throughput transcriptome profiling techniques such as microarray hybridizations and RNA-sequencing analyses are costly, and the analysis of resulting data requires advanced bioinformatics capacity and expertise. On the other hand, single-gene analysis via quantitative real-time reverse transcription polymerase chain reaction (singleplex qPCR) can be time-consuming depending on the number of target transcripts. Multiplexed PCR assays could become a valuable alternative to the aforementioned methodology. In brief, multiplex PCR allows the simultaneous quantitation of multiple gene transcripts, thus making gene expression analysis a much faster process compared with singleplex qPCR. This would also facilitate the addition of more biological replicates, with a relatively low expenditure of time and resources compared with high-throughput techniques.

As introduced above, many metabolic and physiological pathways impacted by dietary FM/FO replacement by terrestrial ingredients have already been identified (Sitjà-Bobadilla et al. 2005; Vilhelmsson et al. 2007; Tacchi et al. 2012; Caballero-Solares et al. 2018), although the molecular mechanisms controlling such diet effects are yet to be elucidated. Furthermore, a number of genes have been repeatedly analyzed across studies and established as informative growth-, metabolism-, stress-, and health-related biomarkers (e.g., Atlantic salmon *fadsd5* and *lect2* in Morais et al. (2012) and Xue et al. (2015)). Multiplex PCR panels constructed with well-characterized gene biomarkers may become a useful tool for aquafeed producers in their search for superior formulations with low content in marine ingredients. Concurrently, this analytical technique could be very useful in the fields of fish nutrition and physiology research, as reflected by the body of literature published to date (e.g., Cleveland and Weber 2011; Cleveland et al. 2012; Cleveland and Weber 2013; Kono and Korenaga 2013; Kono et al. 2013; Cleveland and Weber 2015; Manor et al. 2015).

In the present study, we developed and validated two multiplex PCR panels to evaluate the effect of terrestrial feed ingredients on the transcription of physiologically relevant biomarkers genes in the liver of Atlantic salmon.

## Materials and Methods

### Feeding Trial

The liver tissue samples utilized in the study were collected from Atlantic salmon smolts fed for 14 weeks with either a diet based on marine ingredients (i.e., fish meal (FM) and fish oil (FO)), referred to here as MAR; a high animal by-product/high rapeseed oil diet, referred to as ABP; and a high plant protein/high rapeseed oil diet, referred to as VEG. The formulation and nutritional composition of MAR, ABP, and VEG diets were published in previous studies (Caballero-Solares et al. 2017; Beheshti Foroutani et al. 2018; Caballero-Solares et al. 2018). These studies also described the holding and feeding conditions in detail. Briefly, PIT (passive integrated transponder)-tagged Atlantic salmon smolts (initial weight, 179 ± 29 g (mean ± standard deviation)) were allocated to 620-L tanks connected to a flow-through seawater system in the Dr. Joe Brown Aquatic Research Building (JBARB, Ocean Sciences Centre, Memorial University of Newfoundland, Canada). Each dietary treatment was randomly assigned to 4 replicate tanks, with 40 salmon in each tank. Fish were fed the experimental diets to apparent satiation twice a day for 14 weeks, after which 5 individuals/tank were euthanized via immersion in seawater containing 400 mg/L MS-222 (Syndel Laboratories, Vancouver, BC, Canada) and dissected for tissue collection.

Fish handling, euthanasia, and dissection procedures were performed following the guidelines of the Canadian Council of Animal Care (approved Memorial University Institutional Animal Care Protocol 14–71-MR).

### RNA Extraction and Purification

RNA was extracted, DNaseI-treated, and purified from flash-frozen 50–100 mg liver samples following the standard procedures in the laboratory (Xu et al. 2013; Xue et al. 2015; Caballero-Solares et al. 2018). RNA integrity and purity were verified by 1% agarose gel electrophoresis and NanoDrop UV spectrophotometry (Thermo Fisher Scientific, Mississauga, ON, Canada), respectively. For all samples, no signs of RNA degradation were found (i.e., intact 28S and 18S ribosomal RNA bands), and A260/280 and A260/230 ratios were 2.1–2.3 and 1.9–2.4, respectively.

### Multiplex PCR Panel Development

A list of 40 biomarker genes was selected based mainly on the results from microarray experiments previously conducted in the laboratory. Among the selected biomarkers, there were metabolism- and physiology-relevant genes that were previously microarray-identified as responsive to FO replacement by vegetable oils in the liver of Atlantic salmon (Xue et al. 2020). Other genes with similar functions were identified from a microarray study performed on the same liver RNA samples utilized here and published in Caballero-Solares et al. (2018). Singleplex qPCR data used to confirm the microarray results in Caballero-Solares et al. (2018) have been used to validate the gene expression results generated in the present study (see below). Finally, the list also included biomarker genes involved in cell redox homeostasis, detected via microarray transcriptome profiling of primary Atlantic salmon hepatocytes exposed to an exogenous oxidant (tert-Butyl hydroperoxide, tBHP; Xue et al. unpublished).

The multiplex PCR or eXpress Profiling (XP)-PCR technique developed by Beckman Coulter is based on the use of chimeric primers to amplify DNA fragments of different sizes that are subsequently separated by capillary electrophoresis. The multiplexed analysis allows the quantification of the transcript levels of up to ~ 30 genes per panel, including biomarker and normalizer genes. The chimeric reverse and forward primers consist of a 5′-end universal sequence (5′-GTACGACTCACTATAGGGA-3′ for the reverse, 5′-AGGTGACACTATAGAATA-3′ for the forward), followed by a 3′-end gene-specific sequence. The gene-specific priming sequences were designed to amplify DNA fragments between 137 and 387 bp, with lengths differing by at least 4 bp. For the multiplex analysis, 25 ng of liver RNA template were reverse transcribed in 10 μL of reaction mixture containing 2.5 μL of *kanamycin-resistance* (*kanr*) gene RNA (internal control, pre-diluted 1:8 in 10 mM Tris-HCl, pH 8), 0.5 μL of reverse transcriptase (RT; 10 U), and 2 μL of 5X RT buffer from the GenomeLab™ GeXP Start Kit (Beckman Coulter/SCIEX), as well as 1 μL of chimeric reverse primer mix. Subsequently, 4.65 μL of cDNA were added to the PCR reaction mixture composed of 2 μL 5X PCR buffer from the GenomeLab™ GeXP Start Kit, 0.35 μL Thermo-Start Taq DNA Polymerase (Thermo Fisher Scientific), 2 μL of 25 mM MgCl_2_, and 1 μL of chimeric forward primer mix. RT and PCR reactions were carried out in a DNA Engine Tetrad 2 Peltier thermal cycler (Bio-Rad Laboratories), under the following programs: for the RT, 48 °C for 1 min, then 42 °C for 60 min, and finally 95 °C for 5 min; for the PCR, 1 cycle of 95 °C for 15 min, followed by 35 cycles of 94 °C for 30 s, 55 °C for 30 s, and 70 °C for 1 min. Besides the necessary substrates and salts for the reactions, the 5X RT and PCR buffers contained the reverse and forward primers (also chimeric) for *kanr*. Also, the 5X PCR buffer contained universal WellRED D4 dye-labeled forward and unlabeled reverse primers (homologous to the 5′-ends of the chimeric primers). After the first two PCR cycles, the amplification was taken over by the universal primers as they become predominant in the reaction mixture. Thus, the PCR yielded D4-labeled DNA fragments of different lengths, each length corresponding to a different gene. Once the PCR was concluded, the resultant solution was diluted 1:10 in 10 mM Tris-HCl, pH 8. A loading solution was thereafter prepared by mixing 1 μL of diluted PCR solution with 38.5 μL Sample Loading Solution and 0.25 μL of DNA Size Standard-400 from the GenomeLab™ GeXP Start Kit. Finally, the PCR fragments in the loading solution were separated by capillary polyacrylamide electrophoresis (Frag-3 protocol), and their fluorescence were measured using the GenomeLab™ GeXP genetic analysis system (Beckman Coulter/SCIEX).

We created 2 multiplex PCR panels that included a total of 40 genes and shared 3 normalizer genes. Primers were designed using Primer 3 v.0.4.0 software (available at (http://bioinfo.ut.ee/primer3-0.4.0/)) and quality-checked for PCR fragment size and undesired fragments. The concentration of the reverse primers was adjusted to attenuate the fluorescent signal of the highly abundant transcripts. The linear range of fluorescence detection of the GenomeLab™ GeXP genetic analysis system lies between 370 and 120,000 relative fluorescence units (rfu); however, the optimum range to measure changes in transcript abundance is 2000–50,000 rfu. The reverse primer concentration optimization, together with the pre-dilution of the resultant PCR solution, served to ensure that the amplicons’ fluorescence levels were within the optimum range and that the analysis was quantitative.

For both primer quality check and reverse primer concentration optimization, we used an RNA reference pool including an equal contribution from 12 liver samples, with 4 biological replicates (one from each quadruplicate tank) for each of the three diet groups. Table 1 shows all primers included in the 2 multiplex panels, as well as the GenBank accession numbers of the sequences used for their design, their amplicon sizes, and their functions based on gene ontology (GO) terms from *Danio rerio* and *Homo sapiens* best BLAST hits (from UniProt Knowledgebase, http://www.uniprot.org/).

**Table 1:** List of gene-specific sequences included in the chimeric primers of multiplex panels I and II

Gene name (gene symbol)^a^	GenBank accession number	Amplicon size (bp)	Gene-specific sequence (5′-3′)	Functional annotation^b^
Multiplex panel I				
*Ferritin middle subunit A* (*ftma*)	BT047216	146	F: GCAGAATAGTCGGAGGAACTTT	Cellular iron ion homeostasis
			R: CTTCGCAATCGTGGTGATAG	
*Hepatocyte growth factor A* (*hgfa*)	NM_001140139	151	F: GGCCGCCACCTCTACCTAACGTC	Liver development
			R: CCTTGGCCACTGATATCCTC	
*Choline-phosphate cytidylyltransferase 1 alpha A* (*pcyt1aa*)	BT045986	164	F: ACAAGTTCAAGGGGTTCACG	Phospholipid biosynthetic process
			R: TGCTTGGAGAGGAACTCTGG	
*Glucokinase* (*gck*)	XM_014171080^c^	168	F: CTTTGGAGCCAACGGAGA	Glycolytic process
			R: GCACCAGCTCTCCCATGTA	
*Cytochrome P450 1A1 A* (*cyp1a1a*)	BT045666	172	F: GGTGGGAATGACTCGTACTC	Response to xenobiotic stimulus
			R: GATGTATCCTTGACTGTGCAGT	
*Catalase A* (*cata*)	BT059457	176	F: GAACGGGGTTCAGACCCTACT	Response to oxidative stress
			R: GGACGGTAAGTGCAACAGGT	
*Nuclear factor (erythroid 2-related) factor 2 B* (*nfe2l2b*)	BT044699	186	F: GAGAACATCACGGAGCTGGAAT	Response to oxidative stress
			R: CTCAGCAGACGGAAAACCTC	
*Serine protease HTRA1 B* (*htra1b*)	EG886260	200	F: CAATCAGACTTCCCCGATGT	Regulation of p38MAPK cascade
			R: CTTCCCTCTTGATGGAGCTG	
*Sterol regulatory element-binding protein 1* (*srebp1*)	HM561860	219	F: CCCCAGTTTATCAAGGCTGA	Lipid metabolic process
			R: TCCATCATCACTGGCACTGT	
*Peroxiredoxin 1 B* (*prdx1b*)	NM_001140823	223	F: ACCACCATGTCTGCTGGAAA	Response to oxidative stress
			R: GTCACTGAAGGCCACGATC	
*Apolipoprotein A-I* (*apoaI*)	NM_001141140	230	F: TCTCCCTCCCTTCTCACTCA	Lipoprotein metabolic process
			R: GGCCCTGAACACAGTTCAAA	
*Liver X receptor* (*lxr*)	FJ470290	247	F: GCCGCCGCTATCTGAAATCTG	Steroid hormone receptor activity
			R: CAATCCGGCAACCAATCTGTAGG	
*Insulin-like growth factor binding protein 5b1* (*igfbp-5b1*)	JX565556	252	F: CTGGGTGCTTGGGCTCATATGTT	Regulation of cell growth
			R: CTTCTCTTCTCCATTTCGCG	
*Adenylosuccinate synthetase isozyme 1 C-A* (*adssl1a*)	NM_001139706	257	F: CCAGGTTTGTTTGAGGAGGC	‘de novo*’* AMP biosynthetic process
			R: GTCCTATGCGAGATGCCTTG	
*Elongation of very long-chain fatty acids protein 2* (*elovl2*)	FJ237532	282	F: TGTCCACAATACCCTCCATGC	Fatty acid elongation
			R: GCGACTGGACTTGATGGATT	
*Forkhead box protein A2* (*foxa2*)	CA037594	291	F: CCGGAGCACCACTATTCATT	Cell differentiation
			R: TTCAAGACGGGTTCACGATT	
*Heme oxygenase B* (*hmox1b*)	BT046987	314	F: CTGATGCTGGCCTACCAGAG	Iron ion homeostasis
			R: TGTCTTTGCCGATCTGTCTG	
*Delta 5 fatty acyl desaturase* (*fadsd5*)	AF478472	351	F: GGCAGCAGTGAATGGGGAT	Unsaturated fatty acid biosynthetic process
			R: GAGGCGATCAGCTTGAGAAA	
Multiplex panel II				
*Arachidonate 5-lipoxygenase A* (*5loxa*, also *alox5a*)	NM_001139832	138	F: GCCTCTGCTCACCATGCTGCTGTC	Inflammatory response
			R: TTTTGTGTGGGAGGAGGCTTCC	
*Mechanistic target of rapamycin kinase* (*mtor*)	BT072258	143	F: CTACGACCCACTGCTCAACTG	Regulation of cell growth
			R: TGCTTCGACTGATTGTCCAG	
*Peroxisome proliferator-activated receptor beta/delta A* (*pparba* or *pparda*)	NM_001123635	149	F: CAGCTGATCAACGGTACGAC	Lipid metabolic process
			R: TGCTCTTGGCAAACTCAGTG	
*Lipocalin-type prostaglandin D synthase* (*pgds* or *ptgds*)	BT048787	154	F: TGAGGTGCTCAACAAGCTCTACA	Prostaglandin biosynthetic process
			R: GCAGGAAAGCGATGTTGTCA	
*Fatty acid-binding protein 3 A* (*fabp3a*)	AY509548	165	F: ATAACGATAGACGGTGGTAAGATG	Long-chain fatty acid transport
			R: TGTGGAGACGACGTCACCCAGAGT	
*Cytochrome P450 3A27 B* (*cyp3a27b*)	BT056998	172	F: GCTGTTTGATGCATTGTCCTT	Oxidation-reduction process
			R: TTCAGCAGGTTAGCAGAGTGCC	
*Glucocorticoid receptor* or *nuclear receptor subfamily 3, group C, member 1* (*nr3c1*)	GQ179974	177	F: TTGTGAGGCTGCAGGTGTCTTATGA	Glucocorticoid mediated signaling pathway
			R: CTTCCCCAGCTCCTTTATGTA	
*Succinate dehydrogenase [ubiquinone] iron-sulfur subunit A* (*sdhba*)	EG792456	182	F: GTAGCACCAGCTGCCCTAGTT	Aerobic respiration
			R: AGAGAGAAGGGGTCCTGGAG	
*Heat shock protein 70 A* (*hsp70a*)	BT043589	187	F: GTGGTGAGCGATGGTGGCAA	Protein stabilization
			R: GACAGCATTGTTCACTGGCTT	
*Biliverdin reductase A1* (*blvra1*)	EG875285	192	F: GTGGTTGAACACAGCTCACG	Oxidation-reduction process
			R: TGAACATGCAAGGATCGGTA	
*Immunoglobulin mu heavy chain B* (*igmb*)	BT059185	198	F: CGTGTTTAAGAACAAAGCTGGA	Antibacterial humoral response
			R: CTTTGACATCGCACACAAGC	
*Metallothionein B* (*mtb*)	BT059884	207	F: AAAGAAGCGCGATCAAAAAC	Response to metal ion
			R: CACACAGCCTGAAGCACATT	
*Diacylglycerol O-acyltransferase 2 B* (*dgat2b*)	EG878494	222	F: CGCACTTATTGTGGGGTTCT	Triglyceride biosynthetic process
			R: TCAACATTTCACCACATGGAA	
*Isopentenyl-diphosphate delta-isomerase 1* (*idi1*)	DY710563	240	F: AGACAGCAAAAAGAACTGCCACCTC	Cholesterol biosynthetic process
			R: CCTCTAGCTCCTCCAGCTCA	
*Thioredoxin reductase 1 B* (*txnrd1b*)	GE767612	256	F: CACAAATTGAAGCTGCCAAG	Cell redox homeostasis
			R: ATCCAACTCCACCACATTGC	
*6-phosphofructo-2-kinase/fructose-2,6-biphosphatase 4-like* (*pfkfb4*)	BT044025	264	F: CAACACAACACGGGAGAGGA	Positive regulation of glycolytic process
			R: CGTCCAAAGGCACATAGGTT	
*Sterol regulatory element-binding protein 2* (*srebp2*)	HM561861	290	F: AAGGAGGGGGAGAGGAGAAC	Lipid metabolic process
			R: TCTCCACATCGTCAGACAGC	
*Leukocyte cell-derived chemotaxin-2-like B* (*lect2b*)	DV106130	305	F: CGAAAGCATCCTCTCAGGCT	Response to bacterium
			R: CACTTTGCCGTTGAGTTTCA	
*Apoptosis regulator Bcl2-like 1* (*bcl2l1*)	NM_001141086	311	F: CTTGGTGGGAAGGATCACAG	Hepatocyte apoptotic process
			R: AAGCAATTCCTTCATTTCCCTA	
*Sestrin-1 A* (*sesn1a*)	DY710532	327	F: CTCTGAGAGCGATCACCTGC	Cellular oxidant detoxification
			R: ACCAACAAAGCGCATCTTTC	
*Vitellogenin* (*vtg*)	DY704082	335	F: AAGCCACCTCCAATGTCATC	Cellular response to estrogen stimulus
			R: TCTGCACCCCAAGCAATCTT	
*Thioredoxin A* (*txna*)	BT057544	353	F: GGATGACTTTCTCAATGCCC	Cell redox homeostasis
			R: CCACGTTACTGTTCTCTGGT	
Normalizer genes				
*60S ribosomal protein 32* (*rpl32*)	BT043656	159	F: CGCAGGCGGTTTAAGGGTCAGAT	Translation
			R: TCGAGCTCCTTGATGTTGTG	
*Elongation factor 1 alpha-1* (*eef1a1*)	AF321836	235	F: CTGGCACTTTCACTGCTCAAG	Translational elongation
			R: CAACAATAGCAGCGTCTCCA	
*Beta-actin* (*actb*)	BG933897	297	F: GACGAGGCTCAGAGCAAGAG	Cytoskeleton
			R: AGGGACAACACTGCCTGGAT	

^a^Gene abbreviations are according to UniProt terminology

^b^Genes were functionally annotated based on selected gene ontology (GO) terms from *Homo sapiens* putative orthologues. GO terms were taken from UniProt Knowledgebase

^c^A predicted *Salmo salar gck* DNA sequence was used for primer design as no EST sequence could be found in NCBI databases

### Gene Expression Analysis

Individuals can exhibit different performance under the same dietary conditions, often due to variable food consumption between individuals within a tank. To minimize the impact of biological variability on our analyses, we selected samples from those fish with weight gains within one standard deviation below and above the tank mean value. The multiplex experiment included liver RNA samples of 30 individual fish (i.e., 10 MAR-, 9 ABP-, and 11 VEG-fed fish). Both multiplex panels were run on each sample as well as a no-template control in technical triplicates.

The resultant electropherograms were analyzed using the Fragment Analysis Module of the GenomeLab GeXP genetic analysis system software (Beckman Coulter/SCIEX). The obtained areas under each gene peak were thereafter normalized to *kanr* using the eXpress Profiler software (Beckman Coulter/SCIEX). This normalization step is intended to reduce intercapillary variation. The *kanr*-normalized signal levels were log_2_-transformed and analyzed using geNorm (qBASE plus, Biogazelle) to select the most stable normalizer genes. According to the M values calculated by geNorm, the most stable normalizer genes were *eef1a1* (*M* = 0.213) and *rpl32* (*M* = 0.250).

The relative quantity (RQ) of each transcript was calculated relative to a 7-point standard curve prepared with 1:2 dilutions series of a 40 ng/μL liver RNA reference pool and normalized to the geometric mean of *eef1a1* and *rpl32*. The standard curve was also used to confirm that the amplification conditions (e.g., cDNA input, reverse primer concentration) were not limiting during the 35-cycle PCR analysis of the individual samples.

### Data Transformation and Statistical Analyses

The RQ values of the different biomarker genes analyzed were log_2_-transformed to meet the normality assumption (checked by Kolmogorov-Smirnov test). The log_2_-transformed RQs (log_2_ RQs) were subsequently checked for outliers using Grubb’s test and analyzed through general linear models (GLM) for differences among dietary groups. Any tank-based effect was accounted for by including a random *Tank* factor in the model. If variances were statistically equal among dietary groups (Levene’s test for compliance with the homoscedasticity assumption), the mean values of the three dietary treatments were pairwise compared using Tukey’s post hoc test. Otherwise, if the homoscedasticity assumption was not satisfied, Games-Howell test was used for comparisons. The accepted level of significance was *p* < 0.05. These statistical analyses were conducted using IBM SPSS Statistics v24.0.0 (IBM Corp, Armonk, NY).

For validation purposes, we contrasted, by linear regression analysis, the present multiplex results with previously published singleplex qPCR data (Caballero-Solares et al. 2018) obtained from the same liver RNA samples. This group of singleplex qPCR-analyzed transcripts included *gck*, *pfkfb4*, *adssl1a*, *htra1b*, *idi1*, *fabp3a*, *dgat2b*, *igmb*, *lect2b, eef1a1*, and *rpl32*. For the regression analysis, fold-change values (FCs) were calculated for each dataset (i.e., singleplex and multiplex) using the formula 2^A−B^, where *A* and *B* are the mean log_2_ RQs of two dietary groups (Hori et al. 2012). For down-regulated genes, fold-change values were inverted (− 1/fold change). The significance of the regression model in predicting singleplex FCs using multiplex FCs was determined by *F* test. A significant correlation between both datasets was considered as proof of the validation. The linear regression analysis was performed using IBM SPSS Statistics v24.0.0.

Log_2_-transformed multiplex RQs were also analyzed using principal coordinates analysis (PCoA) for similarities among the different gene expression patterns observed among the salmon. We also tested for differences among gene expression patterns via permutational multivariate analysis of variance (PERMANOVA). PERMANOVA was performed with 9999 random permutations and *Tank* factor nested within the fixed factor *Diet*. The accepted level of significance was *p* < 0.05. Both PERMANOVA and PCoA were based on Bray-Curtis similarities of all pairwise comparisons among individuals. These analyses were performed using PRIMER 6.1.15 (Ivybridge, UK).

We also analyzed the relationships between liver lipid composition and the gene expression data by linear regression analysis. For the regression analysis, we only selected those ω3 and ω6 fatty acids (FAs) accounting for at least 0.5% of the total FAs in the liver. Fish weight gain and other relevant phenotypic parameters (e.g., condition factor, EPA/ARA ratio) were also included. Some of these data were previously published elsewhere (Beheshti Foroutani et al. 2018; Caballero-Solares et al. 2018). The Pearson correlation coefficients (*r*) resulting from the regression analyses were arranged as a correlation matrix. The different genes and phenotypic parameters were grouped and ordered by complete-linkage hierarchical clustering using *r* values. The correlation matrix was generated using IBM SPSS Statistics v24.0.0 and the hierarchical clustering using PRIMER 6.1.15. Liver samples for lipid composition determination were collected, processed, and analyzed as described in Hixson et al. (2017) and Beheshti Foroutani et al. (2018).

## Results

### Validation of Multiplex Gene Expression Data

The linear regression analysis revealed a significant correlation (*F* test, *p* < 0.0001) between multiplex and singleplex fold changes, with an *r*^*2*^ of 0.705 (see Supplementary Fig. S1).

### Glucose Metabolism and Cell Growth-Related Genes

**Fig. 1:**
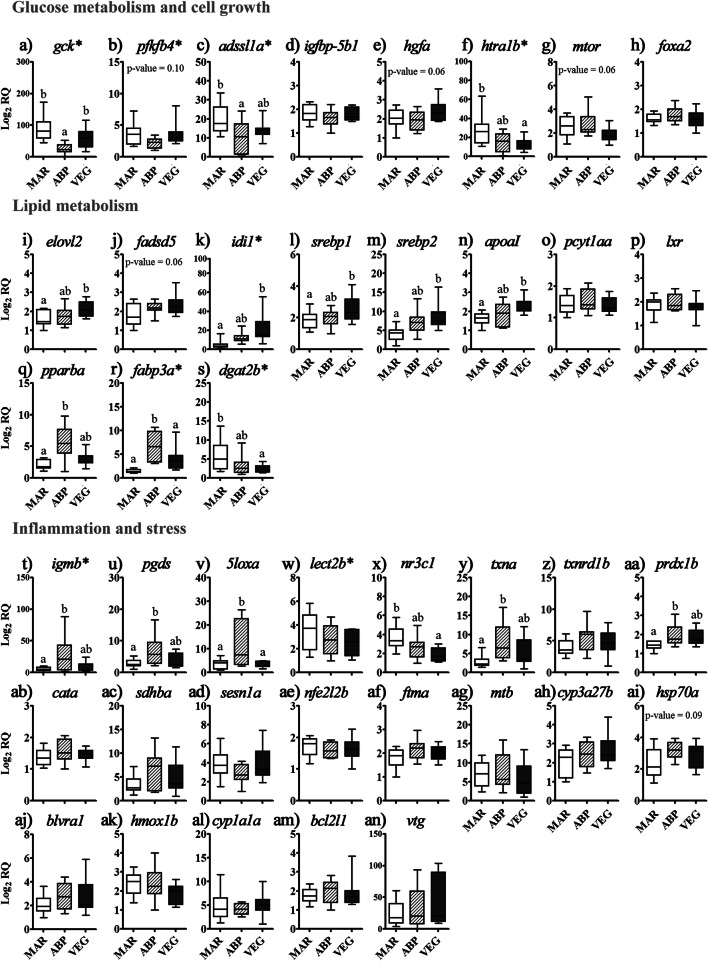
Results from the multiplex PCR analysis of transcripts related to glucose metabolism (**a**–**b**), cell growth (**c**–**h**), lipid metabolism (**i**–**s**), inflammation (**t**–**x**), and stress (**y**–**an**). Boxplots represent relative quantity (log_2_ RQ) medians (horizontal line), interquartile ranges (box), and minimum and maximum values (whiskers). Different letters above upper whiskers represent significant differences between diets (general linear models, Tukey’s (homogeneity of variances among groups) or Games-Howell (variances not homogenous across groups) post hoc test, *p* < 0.05). Transcripts previously analyzed via singleplex qPCR in Caballero-Solares et al. (2018) are indicated with an asterisk after the gene symbol. **a** *glucokinase*; **b** *6-phosphofructo-2-kinase/fructose-2,6-biphosphatase 4-like*; **c** *adenylosuccinate synthetase isozyme 1 C-A*; **d** *insulin-like growth factor binding protein 5b1*; **e** *hepatocyte growth factor A*; **f** *serine protease HTRA1 B*; **g** *mechanistic target of rapamycin kinase*; **h** *forkhead box protein A2*; **i** *elongation of very long chain fatty acids protein 2*; **j** *delta 5 fatty acyl desaturase*; **k** *isopentenyl-diphosphate delta-isomerase 1*; **l** *sterol regulatory element-binding protein 1*; **m** *sterol regulatory element-binding protein 2*; **n** *apolipoprotein A-I*; **o** *choline-phosphate cytidylyltransferase 1 alpha A*; **p** *liver X receptor*; **q** *peroxisome proliferator-activated receptor beta/delta A*; **r** *fatty acid-binding protein 3 A*; **s** *diacylglycerol O-acyltransferase 2 B*; **t** *immunoglobulin mu heavy chain*; **u** *lipocalin-type prostaglandin D synthase*; **v** *arachidonate 5-lipoxygenase A*; **w** *leukocyte cell-derived chemotaxin-2-like B*; **x** *glucocorticoid receptor*; **y** *thioredoxin A*; **z** *thioredoxin reductase 1 B*; **aa** *peroxiredoxin 1 B*; **ab** *catalase A*; **ac** *succinate dehydrogenase [ubiquinone] iron-sulfur subunit A*; **ad** *sestrin-1 A*; **ae** *nuclear factor (erythroid 2-related) factor 2 B*; **af** *ferritin middle subunit A*; **ag** *metallothionein B*; **ah** *cytochrome P450 3A27 A*; **ai** *heat shock protein 70 A*; **aj** *biliverdin reductase A1*; **ak** *heme oxygenase B*; **al** *cytochrome P450 1A1 A*; **am** *apoptosis regulator Bcl2-like 1*; **an** *vitellogenin*

ABP diet down-regulated significantly the hepatic *gck* mRNA levels as compared with MAR and VEG diets (*p* < 0.05, Fig. 1a). The transcript levels of *pfkfb4* followed a similar trend, but differences among diets were not statistically significant (*p* = 0.10, Fig. 1b). Atlantic salmon fed ABP diet also down-regulated *adssl1a* transcription compared with those fed MAR diet (Fig. 1c). VEG diet repressed *htra1b* transcription compared with MAR diet (Fig. 1f). Although not statistically significant (*p* = 0.06), the transcript levels of *hgfa* and *mtor* were, respectively, up- and down-regulated in the liver of salmon fed VEG diet (Fig. 1e, g). Diet did not affect *igfbp-5b1* and *foxa2* transcription (*p* > 0.05, Fig. 1d, h).

### Lipid Metabolism-Related Genes

The transcript levels of *elovl2*, *idi1*, *srebp1*, *srebp2*, and *apoaI* were up-regulated by VEG diet compared with MAR diet (Fig. 1i, k–n). A similar trend was observed for *fadsd5* mRNA levels (Fig. 1j); however, differences among diets were not statistically significant (*p* = 0.06). Conversely, VEG diet down-regulated *dgat2b* transcript levels compared with MAR diet (Fig. 1s). On the other hand, ABP diet increased *pparba* transcription compared with MAR diet (Fig. 1q) and that of *fabp3a* compared with both MAR and VEG diets (Fig. 1r). The mRNA levels of *pcyt1aa* and *lxr* were not dietarily modulated (Fig. 1o, p).

### Inflammation and Oxidative Stress-Related Genes

ABP diet increased the hepatic transcript levels of *igmb*, *pgds*, *txna*, and *prdx1b* compared with MAR diet (Fig. 1t, u, y, aa) and of *5loxa* compared with MAR and VEG diets (Fig. 1v). The mRNA levels of *hsp70a* exhibited a similar trend but were not significantly different (*p* = 0.09, Fig. 1ai). In contrast, *nr3c1* transcript levels in fish fed VEG diet were lower than in those fed MAR diet (Fig. 1x). The other transcripts (*lect2b*, *txnrd1b*, *cata*, *sdhba*, *sesn1a*, *nfe2l2b*, *ftma*, *mtb*, *cyp3a27b*, *blvra1*, *hmox1b*, *cyp1a1a*, *bcl2l1*, and *vtg*; Fig. 1w, z, ab–ah, aj–an, respectively) were not affected by diet.

### Multivariate Analyses—Similarities/Dissimilarities Among Dietary Groups

**Fig. 2:**
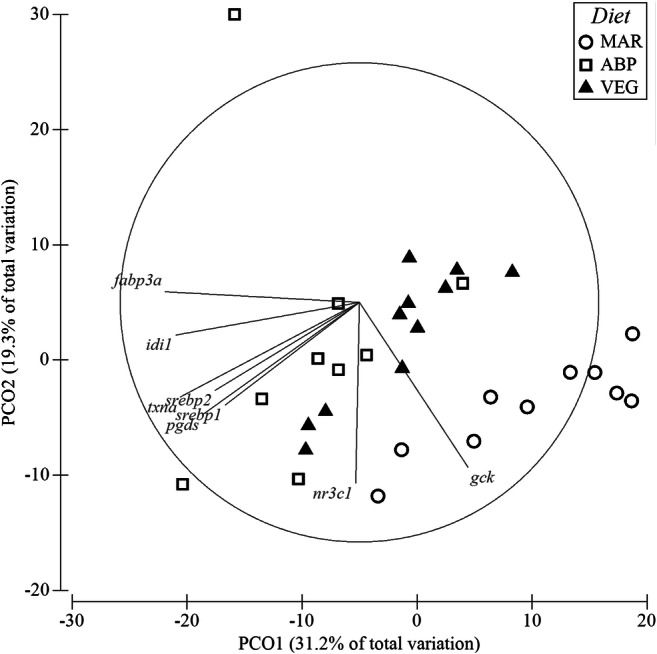
Principal coordinates analysis (PCoA) of the resemblance of salmon fed the different diets based on their gene expression patterns. Vectors indicate the association (Pearson correlation) of a given biomarker gene with the coordinates (axes x and y). Only vectors with Pearson correlation coefficients > 0.7 are shown

The PCoA of the resemblance among salmon based on the multiplex data explained 50.5% of the variation and separated ABP-, VEG-, and MAR-fed salmon—from the top left to the bottom right corner, respectively (Fig. 2). This segregation highly correlated (*r* ≥ 0.70) with *fabp3a*, *idi1*, *txna*, *srebp1*, *srebp2*, *pgds*, *nr3c1*, and *gck*. PERMANOVA found significant differences between fish fed the MAR diet and those fed the ABP or VEG diets (*p* = 0.004–0.008) and close to a significant difference between ABP- and VEG-fed fish (*p* = 0.07).

### Multivariate Analyses—Correlation among Transcriptomic and Phenotypic Parameters

**Fig. 3:**
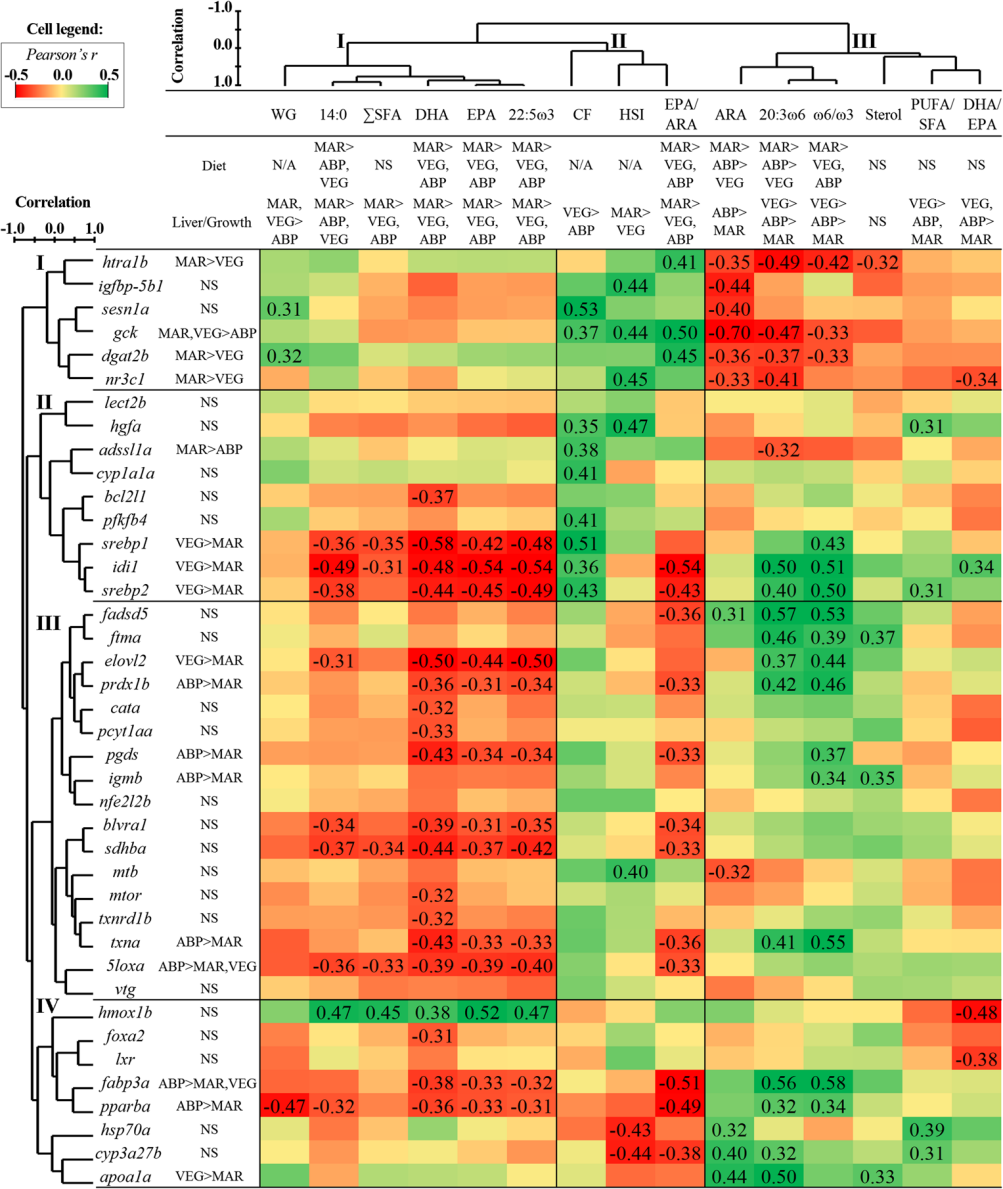
Matrix of Pearson’s correlation coefficients (*r*) between all the analyzed biomarker genes (rows) and a selection of phenotypic features (columns). Significant correlations are indicated by displaying the *r* values in the cell. The different parameters were grouped by complete-linkage hierarchical clustering (using *r* values) and are represented by dendrograms next to gene symbols and above phenotypic parameters. As a reference to the correlated datasets and the dietary context, the significant differences among diet groups are indicated on the right of the gene symbols for the gene expression data and below the phenotypic parameters for the diet composition (“Diet” row) and the liver/growth-related data (“Liver/Growth” row) (general linear models, Tukey’s (homogeneity of variances among groups) or Games-Howell (variances not homogenous across groups) post hoc test; *p* < 0.05). Significant differences are expressed as A > B, A > B,C, or A,B > C; where A, B, and C represent the parameter’s average value in the different diets or dietary groups, and the symbol > means “greater than”. WG, weight gain; SFA, saturated fatty acids; DHA, docosahexaenoic acid (22:6ω3); EPA, eicosapentaenoic acid (20:5ω3); CF, condition factor (body mass/length^3^); HSI, hepatosomatic index [100 × (liver mass/body mass)]; ARA, arachidonic acid (20:4ω6); PUFA, polyunsaturated fatty acids; N/A, not applicable; NS, non-significant differences

The hierarchical clustering analysis grouped the phenotypic parameters into three major clusters (Fig. 3). Weight gain (WG), 14:0, ∑SFA, and the ω3 FAs (i.e., DHA, EPA, and 22:5ω3) comprised cluster I. Condition factor (CF), hepatosomatic index (HSI), and EPA/ARA were grouped in cluster II. The other parameters, i.e., the ω6 FAs 20:3ω6 and ARA, ω6/ω3, sterol, PUFA/SFA, and DHA/EPA, comprised cluster III. Correlations among the phenotypic parameters are shown in Supplemental Fig. S2. With respect to the biomarker genes, four major clusters could be identified by hierarchical clustering (Fig. 3). The gene clusters showed no clear evidence of grouping by function (for gene-to-gene correlations information, see Supplemental Fig. S3). Instead, gene clusters showed different patterns of correlation with the phenotypic parameters. Genes in cluster I distinctively correlated negatively with ARA (significant for all), 20:3ω6 (significant for all except *igfbp-5b1* and *sesn1a*), and ω6/ω3 (significant for *htra1b*, *gck*, and *dgat2b*) and showed positive correlation with WG (significant for *sesn1a* and *dgat2b*) and EPA/ARA (significant for *htra1b*, *gck*, and *dgat2b*). Conversely, genes in clusters II, III, and IV tended to correlate negatively with EPA/ARA and positively with 20:3ω6 and ω6/ω3. These gene-to-trait alignments were clearer for clusters III and IV, based on the higher number of significant correlations. Except for *lect2b* and *bcl21l*, all genes in cluster II showed a significant positive correlation with CF. On the other hand, cluster IV showed a characteristic pattern of negative correlation with CF (tendency, i.e., none was statistically significant), HSI, and EPA/ARA and positive correlation with ARA.

Regardless of the hierarchical clustering analysis, some genes and phenotypic parameters showed correlation patterns that are worth highlighting (Fig. 3). First, several genes in clusters II, III, and IV showed negative correlations with DHA, EPA, and 22:5ω3. Curiously, *hmox1b* in cluster IV deviated from the common pattern and was found to be correlated positively with DHA, EPA, and 22:5ω3. Transcripts related to fatty acid (i.e., *elovl2*, *fabp3a*, and *pparba*) and cholesterol (i.e., *idi1*, *srebp1*, and *srebp2*) metabolism tended to show negative correlations with SFA and ω3 FAs and positive correlations with ω6 FAs and ω6/ω3. Biomarker genes involved in inflammation (i.e., *pgds*) and redox homeostasis (i.e., *prdx1b* and *txna*) showed the same trend.

## Discussion

Multiplex PCR platforms have demonstrated their potential for research in fish physiology and immunology (Cleveland and Weber 2011; Cleveland et al. 2012; Cleveland and Weber 2013; Kono and Korenaga 2013; Kono et al. 2013; Cleveland and Weber 2015; Manor et al. 2015). In the present study, the two multiplex panels based on previous microarray analyses were capable of detecting changes in multiple pathways and processes that are key for Atlantic salmon growth and welfare. Indeed, the multivariate analyses effectively segregated the different dietary groups, both visually (PCoA) and statistically (PERMANOVA). Moreover, the gene expression results revealed that differences were more pronounced between the marine (MAR) and the terrestrial (ABP and VEG) diets than between the two terrestrial diets, thus indicating that the dietary lipid source dominated over the protein source in the transcriptomic changes observed. Relevant performance/phenotypic differences were found among dietary groups—including ABP vs. VEG—that associated with certain gene expression profiles. These relations and their physiological implications are discussed below.

### Glucose Metabolism and Cell Growth and Proliferation

The liver of Atlantic salmon fed ABP diet showed repressed transcription of genes involved in the glycolytic pathway (*gck* and *pfkfb4*). Our previous microarray and singleplex qPCR results indicated likewise (Caballero-Solares et al. 2018), but here we confirmed the inverse relation between *gck* and ω6 FAs in the liver of salmon. High dietary ω6/ω3 ratios have been associated with insulin resistance in mammals (Storlien et al. 1997; Poudyal et al. 2011). Conversely, ω3 FAs improve glucose utilization and reduce insulin resistance in rats (Poudyal et al. 2011). As reviewed by Poudyal et al. (2011) and Scorletti and Byrne (2013), glycolytic and lipogenic enzymes in mammals are transcriptionally co-regulated by PPARs and SREBP1. Research in fish nutrition revealed that SREBP1a activates the transcription of *gck* and *pfkfb1* in gilthead seabream (Metón et al. 2006; Egea et al. 2008). In contrast to SREBP1, SREBP2 predominantly regulates genes involved in cholesterol homeostasis (Shimomura et al. 1997; Horton et al. 2002; Horton et al. 2003). Here, *srebp1* and *srebp2* transcription was up-regulated by VEG diet, which correlated positively with the higher liver ω6/ω3 ratios and *pfkfb4* (not *gck*) mRNA levels found in salmon fed this diet. However, neither *gck* nor *pfkfb4* showed a positive association with any of the hepatic ω6 FA parameters analyzed. This is largely due to MAR-fed salmon as their livers showed distinctive high *gck* and *pfkfb4* transcript levels and low ARA, 20:3ω6, and ω6/ω3 values. As discussed in Caballero-Solares et al. (2018), MAR diet had a higher carbohydrate content than ABP and VEG, which could have contributed to *gck* and *pfkfb4* up-regulation. Contrary to previous studies reporting that the activation of mammalian PPARB improves glucose utilization through *gck* up-regulation (Liu et al. 2010), our study found *gck* and *pparba* mRNA levels to be negatively correlated. The higher *pparba* mRNA levels in ABP-fed fish were the predominant driver of this relationship. Lower feed intake had previously been argued to be responsible for *gck* and *pfkfb4* down-regulation in the fish fed ABP diet (Caballero-Solares et al. 2018). However, in view of *pparba* positive correlation with liver ω6 FA levels (i.e., 20:3ω6 and ω6/ω3), and likewise for *srebp1* and *srebp2*, the influence of diet-related changes on liver lipid metabolism should also be considered. In summary, the expression patterns observed here for *gck*, *pfkfb4*, *srebp1*, *srebp2*, and *pparba* might reflect the myriad of specific and shared mechanisms ruling fish glucose and lipid metabolism upon different dietary conditions (i.e., diet FA profile, diet carbohydrate content, and feed intake).

The transcription of *gck* correlated positively with that of the cell growth and proliferation-related genes *igfbp-5b1* and *htra1b*. IGFBP5 regulates IGF-I signaling in mammals and fish (Duan and Xu 2005; Macqueen et al. 2013; Cleveland and Weber 2015), and *igfbp-5b1* down-regulation has been associated with a camelina-induced reduction in Atlantic salmon growth (Xue et al. 2015). In the present study, *igfbp-5b1* mRNA levels did not show significant differences among diets nor correlate with WG. Nevertheless, *igfbp-5b1* did correlate positively with *htra1b*, which encodes a protease known to cleave IGFBP5 in mammals (Clausen et al. 2011). Given that *htra1b* transcription was down-regulated in the VEG-fed fish compared with those fed the MAR diet (both in the present study and in Caballero-Solares et al. (2018)), a dietary impact on free IGF-I concentrations (and activity, as discussed in Cleveland and Weber (2015)) can be suspected.

Despite not correlating significantly with WG, *gck*, *igfbp-5b1*, and *hgfa* correlated positively with the increased HSI in salmon fed MAR diet compared with those fed VEG diet. Given WG similarities between MAR and VEG-fed fish, higher HSI in MAR-fed fish could be due to increased deposition of hepatic glycogen (since glucose phosphorylation by GCK initiates the glycogenesis) and/or promotion of cell proliferation. On the other hand, *gck*, *igfbp-5b1*, and *htra1b* mRNA levels were inversely correlated with the hepatic ARA levels, which, in turn, were negatively correlated with HSI. Previous studies found increasing dietary ARA levels to reduce HSI in fish (Xu et al. 2010; Shahkar et al. 2016; Torrecillas et al. 2018). In the present study, although MAR diet had higher ARA content than ABP diet, ABP-fed fish presented higher ARA concentration in the liver. Interestingly, liver ARA levels correlated negatively with *dgat2b* and hence with the synthesis of triacylglycerol. Suppressing *dgat2b* expression reduced hepatic steatosis in rats (Choi et al. 2007), while Torrecillas et al. (2018) linked lower HSI in European sea bass with an ARA-induced reduction of hepatocyte lipid vacuolization. Taken together, our results point to MAR diet promoting liver tissue development and accumulation of energy storage products (i.e., glycogen and lipids).

### Lipid Metabolism

The fatty acid profile of VEG diet seemed to stimulate fatty acid elongation and desaturation, as suggested by *elovl2* and *fadsd5* results (significant up-regulation in *elovl2*; close to significant (*p* = 0.06) in *fadsd5*). Desaturation and elongation of PUFAs by FADSD5 (or FADS2D5; Betancor et al. (2014)) and ELOVL2 have been described in Atlantic salmon (Hastings et al. 2004; Morais et al. 2009), thus demonstrating the enzymatic capacity to synthesize EPA, DHA, and ARA. At the gene expression level, low dietary levels of EPA and DHA induce *elovl2* and *fadsd5* transcription in the liver of Atlantic salmon (Betancor et al. 2014; Xue et al. 2015, 2020). Here, a slight, 1.3 fold induction of *elovl2* (statistically significant) and *fadsd5* (*p* = 0.06) was observed when comparing VEG-fed and MAR-fed salmon, which is close to that found by Hixson et al. (2017) between the fish fed control fish oil (FO)-based and the high-vegetable oil (VO) diets. Also, the differences in EPA and DHA levels between control FO-based and high-VO diets were comparable between the present study and Hixson et al. (2017). Yet changes in hepatic *elovl2* and *fadsd5* mRNA levels were not significant in Hixson et al. (2017), which is likely due to differences in the design of the experiment and the statistical analysis: the present compares 3 diets with contrasted formulations, while Hixson et al. (2017) tested 10 diets with more gradual variation in their FA composition.

As expected, based on previous studies (Turchini et al. 2009; Xue et al. 2020), hepatic EPA and DHA levels reflected those of the diets, which translated into a negative correlation with *elovl2*. In contrast, liver ARA and precursor 20:3ω6 levels correlated positively with *fadsd5*. Increased liver ARA levels in fish fed the VEG, and ABP diets, despite consuming diets with less ARA, are in agreement with previous studies in Atlantic salmon (Tocher et al. 1997; Jordal et al. 2007). Similar to those studies, the high-VO diets in our study were more abundant in precursor linoleic acid (LNA, 18:2ω6) than the FO-based diet. As illustrated in Katan et al. (2019), dietary LNA is largely converted into ARA in the liver of Atlantic salmon. Considering the above, it appears that the higher LNA content of ABP and VEG diets enhanced the synthesis of ARA in the liver of salmon, regardless of dietary ARA concentration. However, the interaction of dietary ARA and ω6/ω3 modulatory effects on Atlantic salmon liver FA metabolism remains unclear.

Liver DHA and EPA showed association with almost half of the analyzed biomarker genes, thus suggesting that most biological processes studied here were affected by these nutrients. Continuing with those involved in lipid metabolism, both DHA and EPA were negatively correlated with the transcription factors *srebp1* and *srebp2*. DHA and EPA were previously reported to down-regulate *srebp1* in Atlantic salmon in vitro (SHK-1 cell line; Minghetti et al. (2011)) and in vivo (liver; Hixson et al. (2017)). In mammals, SREBP1 up-regulates the transcription of genes involved in FA desaturation and elongation (Horton et al. 2003). However, as previously stated, SREBP2 predominantly regulates genes involved in cholesterol biosynthesis (Shimomura et al. 1997; Horton et al. 2002, 2003). To explain the negative correlation with liver EPA and DHA levels shared by *srebp1* and *srebp2*, it should be noted that marine ingredient replacement by plant products jointly affects dietary levels of DHA, EPA, and cholesterol. In fact, the present and previous studies suggest that Atlantic salmon SREBP1 and SREBP2 play similar roles to those of mammals. First, our correlation analyses revealed *fadsd5* and *elovl2* to be more strongly related to *srebp1* than to *srebp2* (*r*, 0.57–0.67 (*srebp1*) vs 0.41–0.42 (*srebp2*)). Second, previous studies showed Atlantic salmon *srebp2* to be induced by low cholesterol/high phytosterol levels in plant-based feeds (Leaver et al. 2008; Kortner et al. 2012; Liland et al. 2013; Kortner et al. 2014). In the present study, *srebp2* mRNA levels were strongly correlated with *idi1* (*r* = 0.81), a transcript encoding an enzyme involved in cholesterol biosynthesis. The correlation between *srebp1* and *idi1* was not as strong (*r* = 0.69). We found no correlation of *srebp1*, *srebp2*, and *idi1* with liver sterol levels. However, lipid class separation by thin-layer chromatography does not discriminate between cholesterol and phytosterols. Therefore, the sterol concentration data presented here may not reflect diet-related changes in cholesterol biosynthesis rates in salmon livers. Finally, VEG diet up-regulated *apoaI* transcription (as compared with MAR diet), which could be interpreted as a promotion of lipoprotein assembly (mainly high-density lipoproteins (HDLs)) and lipid transport. Similar results and explanation were reported by Gu et al. (2013) in their study on the effects of plant meal-based diets on the liver of Atlantic salmon. In support of this hypothesis, *apoaI* mRNA levels correlated positively with liver sterol levels. Since hepatic cholesterol synthesis was also enhanced by VEG, as suggested by *idi1* transcript levels, it would appear that the liver metabolic machinery adapted to lower dietary cholesterol intake to maintain cholesterol levels.

Liver DHA and EPA levels correlated negatively with *pparba* mRNA levels, which were up-regulated by ABP diet compared with MAR diet. In turn, *pparba* up-regulation correlated negatively with WG, as well as with other phenotypic parameters such as 14:0, 22:5ω3, and EPA/ARA. With respect to 14:0, the activation of mammalian PPARB was found to reduce hepatic SFA levels while increasing those of monounsaturated FAs (Liu et al. 2010). As in mammals, Atlantic salmon PPARB has been suggested to up-regulate genes involved in beta-oxidation (Torstensen et al. 2009). Despite the above, and that FA membrane-mitochondria transport seemed to be up-regulated in ABP-fed fish (i.e., higher *fabp3a* transcription compared with MAR), our previous study found no dietary-induced changes in the expression of beta-oxidation-related transcripts (*acyl-coenzyme A oxidase 1* and *carnitine palmitoyltransferase 1*; Caballero-Solares et al. (2018)). Nevertheless, changes in hepatic beta-oxidation activity may not agree with that observed at the transcriptomic level. As inferred from Kjær et al. (2008), damaged mitochondrial function due to oxidative stress is another factor to consider when discussing beta-oxidation in the liver of Atlantic salmon. Indeed, FABP3 overexpression has been associated with mitochondrial dysfunction in murine cells (Song et al. 2012). In this regard, it should be noted that *pparba* and *fabp3a* mRNA levels correlated positively with those of *txna* and *prdx1b*. TXN and PRDX proteins are well known for remediating oxidative stress in mammals (Nordberg and Arnér 2001) and fish (Pacitti et al. 2014). These gene expression patterns suggest interactions between oxidative stress-induced mitochondrial injury and cell apoptosis (i.e., mitochondrial permeability transition) and inflammation (Kowaltowski et al. 2001; Tschopp 2011).

### Inflammation and Stress

We previously presented evidence that ABP diet promoted the transcription of the pro-apoptosis biomarker *growth arrest and DNA damage-inducible protein GADD45 beta* (*gadd45b*; Caballero-Solares et al. 2018). In the present study, the mRNA levels of the anti-apoptosis biomarker *bcl2l1* (Eimon and Ashkenazi 2010) were not significantly affected by diet but correlated significantly and positively with those of *txna* and *prdx1b*, which were significantly up-regulated by ABP diet. Similar expression patterns of *gadd45b* and *bcl2l1* may suggest a tight balance between pro- and anti-apoptosis regulators. With respect to the connection between cellular oxidative stress (i.e., *txna*, *prdx1b*) and apoptosis (i.e., *bcl2l1*), the literature on Atlantic salmon nutrigenomics indicates that specific diet formulations can increase cell oxidative stress and apoptosis. In Kjær et al. (2008), excessive dietary DHA and EPA levels caused mitochondrial membrane disruption, oxidative stress, and increased apoptosis in the liver of Atlantic salmon. Excessive dietary DHA and EPA levels would not have caused the aforementioned gene expression changes, as hepatic DHA and EPA levels were lower in the ABP-fed fish compared with the MAR-fed fish, clearly reflecting diet composition. Consequently, liver DHA levels were inversely proportional to the transcript levels of most oxidative stress (i.e., *prdx1b*, *cata*, *blvra1*, *sdhba*, *txnrd1b*, *txna*) and apoptosis biomarkers (*bcl2l1*). Moreover, DHA and EPA levels in the present experimental diets were 17–55 times lower than in those tested in Kjær et al. (2008). On the other hand, despite its similarity with ABP diet in dietary and hepatic DHA and EPA levels, VEG diet did not elicit such transcriptomic changes, thus discarding FM replacement by plant alternatives as the cause, contrary to previous findings in Atlantic salmon (Tacchi et al. 2012). To our knowledge, the effects of animal by-products in aquafeeds on the hepatic fish transcriptome had not been investigated prior to Caballero-Solares et al. (2018). Given that ABP appeared to reduce feed intake, which is most likely what lowered weight gain (Beheshti Foroutani et al. 2018; Caballero-Solares et al. 2018), this question would be best addressed as in Skugor et al. (2011). Skugor et al. (2011) found that both dietary soybean meal inclusion and feed restriction similarly induced the transcription of pro-apoptotic genes and repressed that of relevant oxidative stress genes in the liver of Atlantic salmon. In mice, caloric restriction is known to increase hepatic apoptosis but—as opposed to our results—decrease oxidative stress (Gredilla and Barja 2005; Spindler and Dhahbi 2007). To this latter contradiction must be added the fact that, while anti-inflammatory properties were attributed to caloric restriction in mice (Cao et al. 2001), the ABP-fed fish in the present study showed increased mRNA levels of various pro-inflammatory biomarker genes (i.e., *5loxa*, *pgds*, and *igmb*). Therefore, there is no substantial evidence for dietary DHA and EPA levels, or feed intake, to have caused the apparent increase in oxidative stress, apoptosis, and inflammation in the liver of the ABP-fed fish. Instead, our correlation data lead our attention to the ω6 FAs.

The pro-inflammatory properties of ABP diet were previously reported (Caballero-Solares et al. 2017, 2018). The present multiplex PCR data served to confirm these previous findings since ABP diet up-regulated the transcription of genes involved in the synthesis of eicosanoids (*5loxa*, *pgds*) and *igmb* (IgM+ B cells were found to drive peritoneal inflammatory response in rainbow trout (Castro et al. 2017; Caballero-Solares et al. 2018)). Furthermore, the up-regulation of all three genes correlated significantly with pro-inflammatory hepatic FA profiles, such as low EPA/ARA and high ω6/ω3 and 20:3ω6 levels. Low hepatic EPA/ARA levels would promote the synthesis of pro-inflammatory eicosanoids (i.e., ARA-derived eicosanoids) by 5LOX and PGDS (Wall et al. 2010). Interestingly, the oxidative stress-related genes *prdx1b* and *txna* showed a similar correlation to these FA parameters. Also, the inflammation-related genes *5loxa*, *pgds*, and *igmb* correlated positively with those representing oxidative stress (i.e., *prdx1b* and *txna*), apoptosis (i.e., *bcl2l1*), and mitochondrial function (i.e., *fabp3a*). A growing body of evidence links ARA to cellular oxidative stress and apoptosis in mammals (Wolf and Laster 1999; Brash 2001; Chen and Chang 2009; Pompeia et al. 2012; Shen et al. 2018). According to the mammalian literature, ARA-induced cell death would result from the depolarization of mitochondria by activation of mitogen-activated protein kinases (p38α MAPK and JNK) and the promotion of reactive oxygen species (ROS) production. In contrast to mammals, to the best of our knowledge, the role of ARA in the regulation of cellular redox homeostasis and cell death has yet to be investigated in fish.

### Conclusions

In the present study, we analyzed the effects of terrestrial feed ingredients on the transcript levels of 40 physiologically relevant biomarker genes in the liver of Atlantic salmon using multiplex PCR assays. The endpoint multiplex PCR assays were optimized to ensure the quantitative accuracy of the analysis, which was validated by correlation analysis with singleplex qPCR data (*r*^2^ = 0.705; *p* < 0.0001). Our multiplex PCR data showed ingredients of terrestrial origin modulated metabolism, growth-related mechanisms, inflammation, and oxidative stress in the liver of Atlantic salmon. Additionally, correlations between transcript abundance and response parameters such as HSI and liver ARA, DHA, and EPA levels indicated physiological impacts of nutrient-gene interactions induced by the terrestrial feed ingredients. For example, the association of DHA, EPA, and ARA with gene biomarkers of oxidative stress and apoptosis suggested terrestrial ingredients may have significant effects on health status. Investigating numerous gene biomarkers using multiplex PCR provided the capacity to quickly and cost-effectively determine how multiple physiological processes respond to dietary modulation. Utilizing these assays in future research will allow us to identify gaps of knowledge in fish physiology (e.g., effects of animal by-products on liver function) and draw hypotheses for future studies (e.g., ARA induction of cell death via oxidative stress).

## Electronic supplementary material

ESM 1 Scatter plot of fold-changes between diets calculated using log_2_-transformed multiplex (x axis) and singleplex (y axis) relative quantity (log_2_ RQ) gene expression values. Each dot represents either an ABP vs MAR, VEG vs MAR, or VEG vs ABP comparison for a given gene. (PNG 124 kb)

High Resolution Image (TIF 61 kb)

ESM 2 Matrix of Pearson’s correlation coefficients (*r*) among the selected phenotypic features. Significant correlations are indicted by displaying the *r* values in the cell. Despite being redundant, half of each correlation matrix was not omitted to facilitate its interpretation. (PNG 380 kb)

High Resolution Image (TIF 245 kb)

ESM 3 Matrix of Pearson’s correlation coefficients (*r*) among the analyzed biomarker genes. Significant correlations are indicted by displaying the *r* values in the cell. Despite being redundant, half of each correlation matrix was not omitted to facilitate its interpretation. (PNG 1447 kb)

High Resolution Image (TIF 1453 kb)

## Data Availability

The raw data supporting the conclusions of this manuscript will be made available by the authors, without undue reservation, to any qualified researcher.
