# Supplementary figures and images for: Diet-Induced Physiological Responses in the Liver of Atlantic Salmon (Salmo salar) Inferred Using Multiplex PCR Platforms
Source: Mar Biotechnol (NY). 2020 Jun 4;22(4):511–25. doi: 10.1007/s10126-020-09972-5 (PMC8346449; doi:10.1007/s10126-020-09972-5)

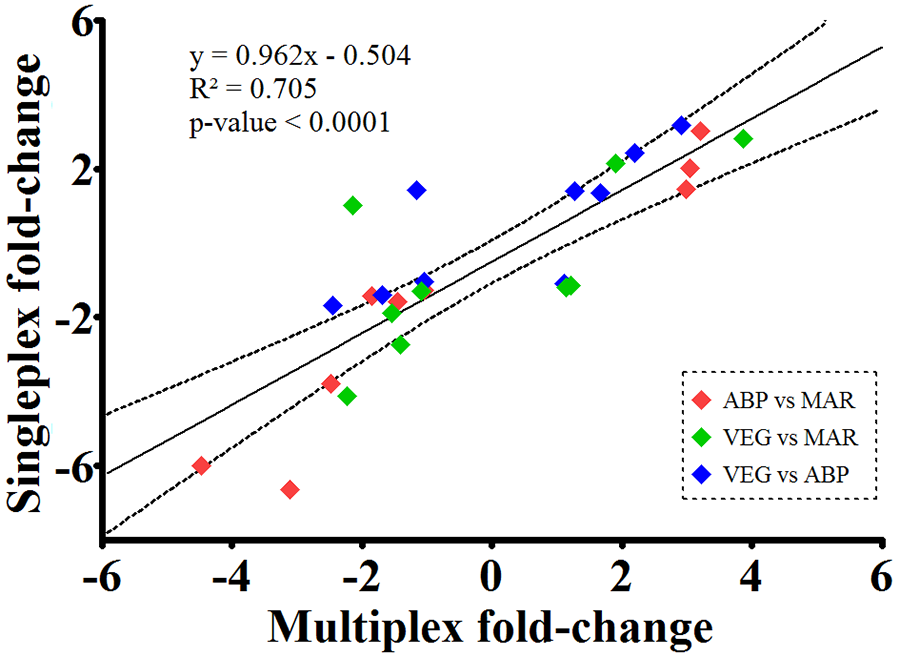

Supplement: Supplementary file 1 — Scatter plot of fold-changes between diets calculated using log2-transformed multiplex (x axis) and singleplex (y axis) relative quantity (log2 RQ) gene expression values. Each dot represents either an ABP vs MAR, VEG vs MAR, or VEG vs ABP comparison for a given gene. (PNG 124 kb) [file 10126_2020_9972_Fig4_ESM.png]

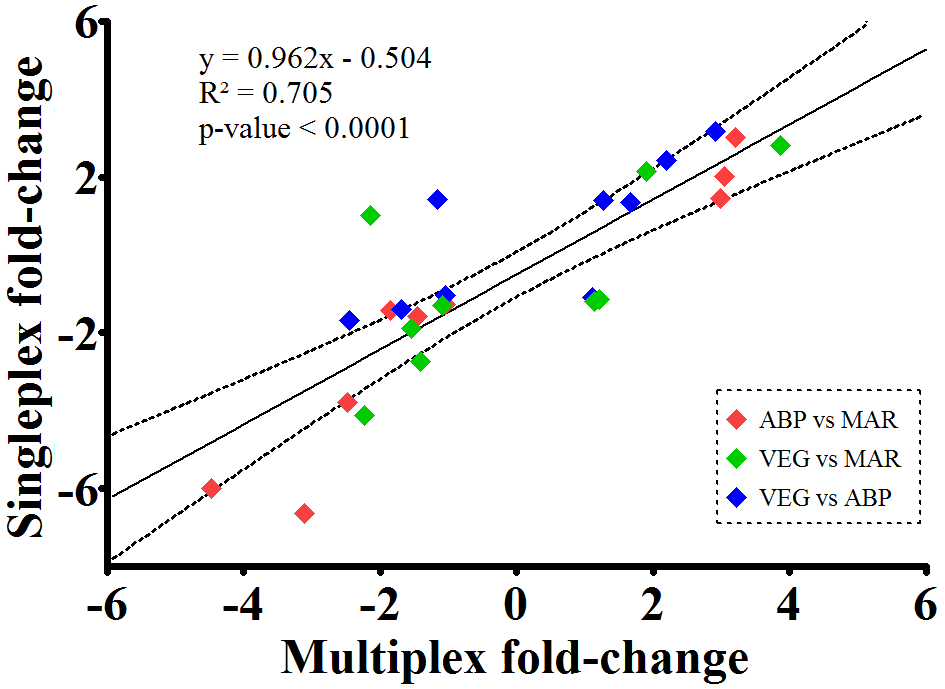

Supplement: Supplementary file 2 — High Resolution Image (TIF 61 kb) [file 10126_2020_9972_MOESM1_ESM.tif]

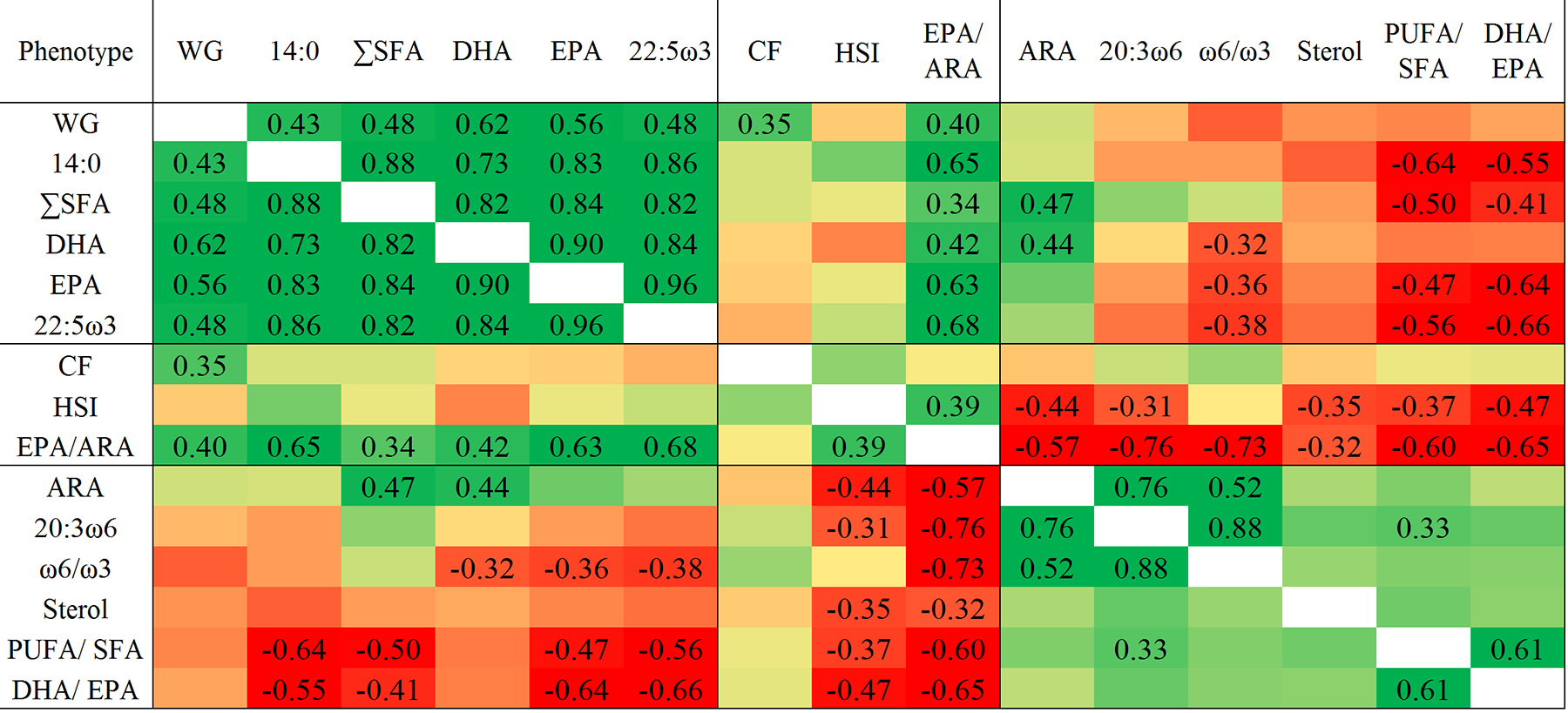

Supplement: Supplementary file 3 — Matrix of Pearson’s correlation coefficients (r) among the selected phenotypic features. Significant correlations are indicted by displaying the r values in the cell. Despite being redundant, half of each correlation matrix was not omitted to facilitate its interpretation. (PNG 380 kb) [file 10126_2020_9972_Fig5_ESM.png]

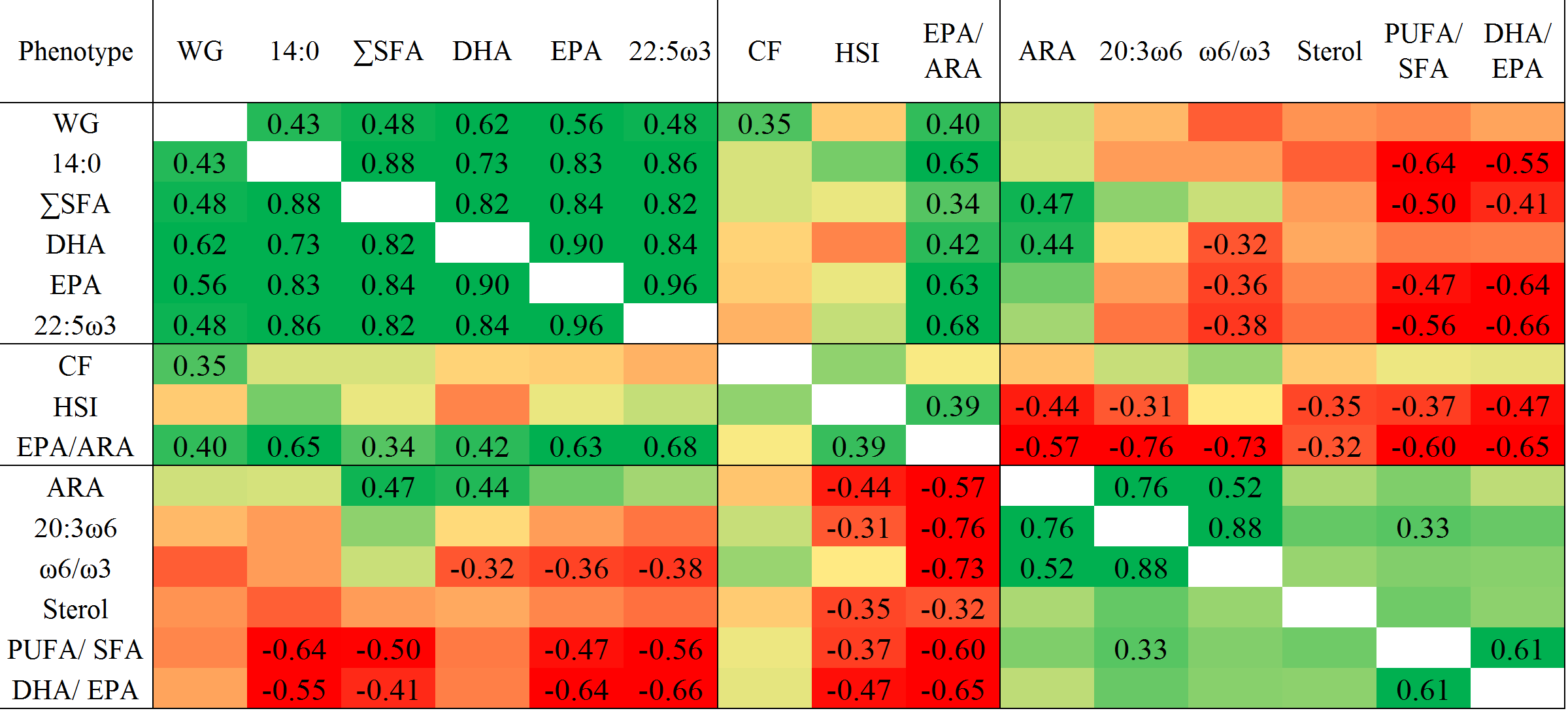

Supplement: Supplementary file 4 — High Resolution Image (TIF 245 kb) [file 10126_2020_9972_MOESM2_ESM.tif]

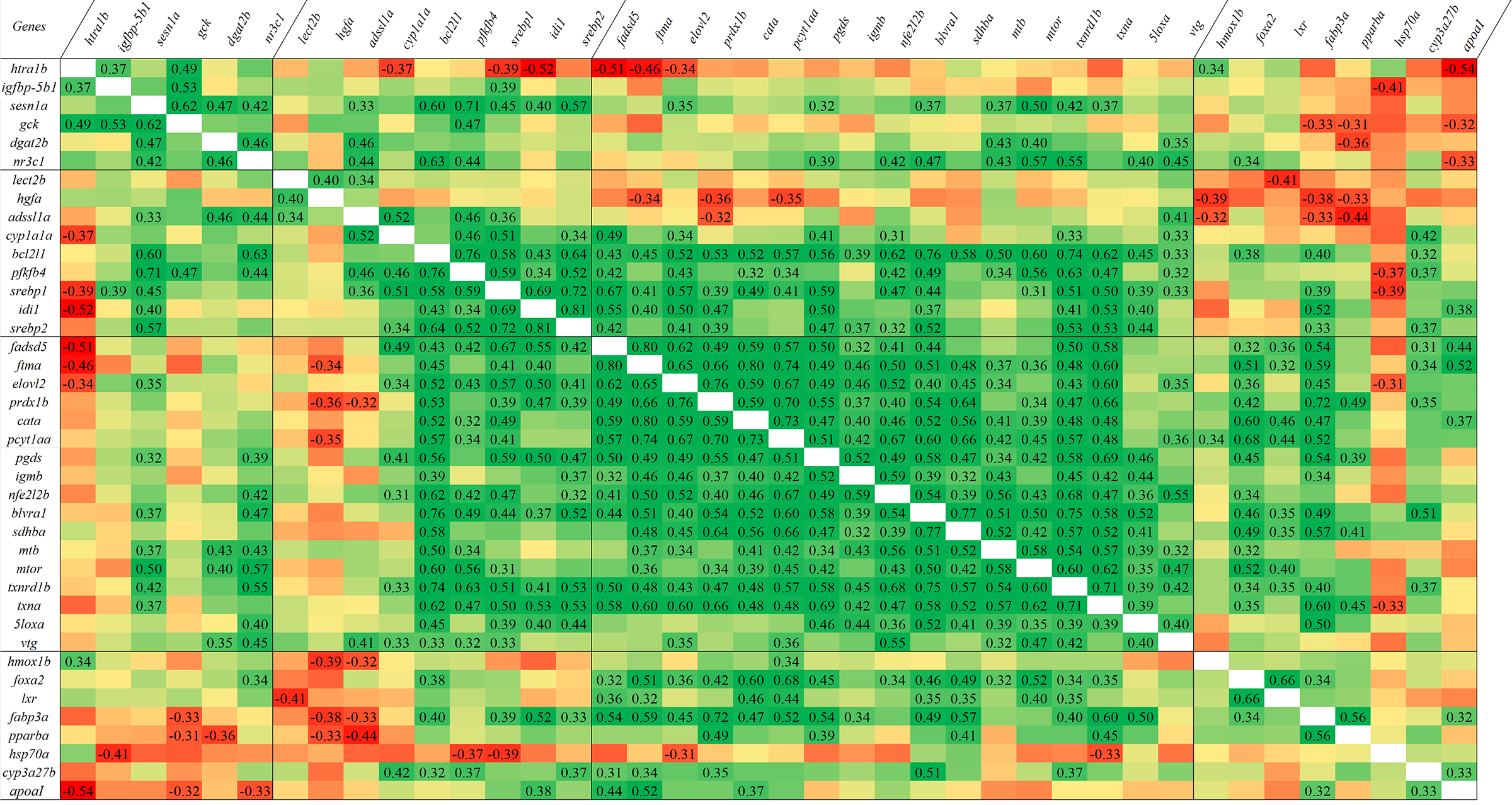

Supplement: Supplementary file 5 — Matrix of Pearson’s correlation coefficients (r) among the analyzed biomarker genes. Significant correlations are indicted by displaying the r values in the cell. Despite being redundant, half of each correlation matrix was not omitted to facilitate its interpretation. (PNG 1447 kb) [file 10126_2020_9972_Fig6_ESM.png]

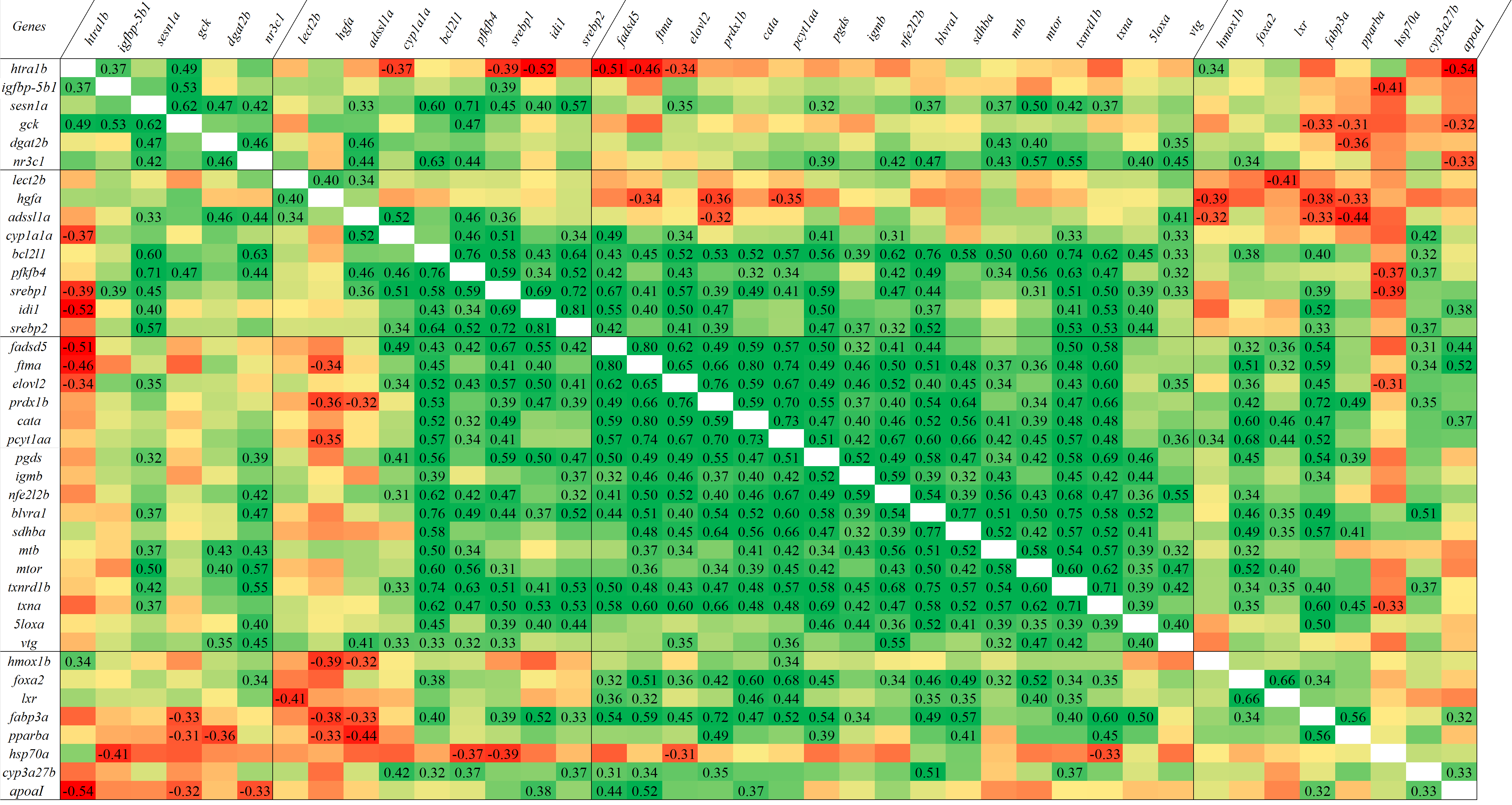

Supplement: Supplementary file 6 — High Resolution Image (TIF 1453 kb) [file 10126_2020_9972_MOESM3_ESM.tif]
